# Lipid Catabolism and ROS in Cancer: A Bidirectional Liaison

**DOI:** 10.3390/cancers13215484

**Published:** 2021-10-31

**Authors:** Serena Castelli, Pamela De Falco, Fabio Ciccarone, Enrico Desideri, Maria Rosa Ciriolo

**Affiliations:** 1 Department of Biology, University of Rome “Tor Vergata”, Via Della Ricerca Scientifica 1, 00133 Rome, Italy; serenacastelli93@gmail.com (S.C.); pameladefalco@hotmail.it (P.D.F.); enrico.desideri@uniroma2.it (E.D.); 2 IRCCS San Raffaele Pisana, Department of Human Sciences and Promotion of the Quality of Life, San Raffaele Roma Open University, 00166 Rome, Italy; fabio.ciccarone@uniroma5.it; 3 IRCCS San Raffaele Pisana, Via Della Pisana 235, 00163 Rome, Italy

**Keywords:** lipid catabolism, reactive oxygen species, fatty acid oxidation, mitochondrial metabolism

## Abstract

**Simple Summary:**

The Warburg effect, the utilization of glycolysis as a source of ATP under normoxia, is one of the main hallmarks of cancer. It is included in a wider concept of the metabolic adaptations of cancer cells, in which mitochondria also have crucial roles. Indeed, mitochondria are responsible for the catabolism of macromolecules, which then feeds oxidative phosphorylation. Among the catabolic pathways, fatty acid oxidation is the most efficient in energy supply, but is harmful due to the unavoidable production of reactive oxygen species (ROS). Therefore, the heterogeneity in cancer’s metabolic adaptations is partly due to the capacity to cope with those pro-oxidant compounds. Herein, we will highlight recent results showing the opposite effects of lipid catabolism on cancer progression depending on intracellular ROS. In parallel, we will analyze the evidence of the regulation of lipid catabolism by ROS. Finally, we will summarize the consequences of the mutual relation of fatty acid oxidation and ROS for therapeutic purposes.

**Abstract:**

Although cancer cell metabolism was mainly considered to rely on glycolysis, with the concomitant impairment of mitochondrial metabolism, it has recently been demonstrated that several tumor types are sustained by oxidative phosphorylation (OXPHOS). In this context, endogenous fatty acids (FAs) deriving from lipolysis or lipophagy are oxidised into the mitochondrion, and are used as a source of energy through OXPHOS. Because the electron transport chain is the main source of ROS, cancer cells relying on fatty acid oxidation (FAO) need to be equipped with antioxidant systems that maintain the ROS levels under the death threshold. In those conditions, ROS can act as second messengers, favouring proliferation and survival. Herein, we highlight the different responses that tumor cells adopt when lipid catabolism is augmented, taking into account the different ROS fates. Many papers have demonstrated that the pro- or anti-tumoral roles of endogenous FA usage are hugely dependent on the tumor type, and on the capacity of cancer cells to maintain redox homeostasis. In light of this, clinical studies have taken advantage of the boosting of lipid catabolism to increase the efficacy of tumor therapy, whereas, in other contexts, antioxidant compounds are useful to reduce the pro-survival effects of ROS deriving from FAO.

## 1. Introduction 

The uncontrolled proliferation of cancer cells is a process strictly associated with a high energy demand to ensure the increased synthesis of macromolecules for cellular building blocks [1]. In order to satisfy these requirements, cancer cells adjust their metabolism to favour glycolysis, the rapid source of energy production from glucose, a process termed the “Warburg effect”. Moreover, the enlargement of the tumor mass creates a harsh environment, in which cancer cells are challenged by the low availability of oxygen and nutrients due to the poor presence of blood vessels, particularly in the inner part of the mass [2]. Metabolic plasticity allows cancer cells to cope with these conditions by favouring lactate fermentation over energy deriving from oxidative phosphorylation (OXPHOS), which requires oxygen. Warburg hypothesized that these metabolic modifications of cancer cells stemmed from the dysfunctionality of mitochondria, which is the first biochemical event starting the cancerous transformation. However, this former picture suggesting OXPHOS impairment/downregulation in cancer has been not corroborated by recent research, which instead highlighted the crucial role of mitochondrial metabolism in producing ATP and metabolites for biosynthetic purposes [3] in many different cancers, including leukaemia [4], lung adenocarcinoma [5] and melanoma [6]. 

The complexity of the metabolic adaptations of cancer cells at the mitochondrial level is further amplified by glutamine, which plays a crucial role in replenishing Tricarboxylic acid (TCA) cycle intermediates, with these being a key cellular carbon source and participating in cell growth, redox balance and energy production [7,8]. Indeed, glutamine deamination provides glutamate, which has different fates in cell metabolism, including protein and glutathione synthesis, transamination and α-ketoglutarate production, which feeds the TCA cycle [9]. 

Other metabolites fueling the Krebs cycle are ketone bodies, among which 3-hydroxy-butyrate and acetoacetate can be efficiently converted in acetyl-CoA. Although in normal conditions, ketone bodies are mainly produced under fasting, some tumors undergo metabolic rewiring aiming to use ketone bodies produced by tumor stroma, particularly by fibroblasts. The acquisition of the capacity to use ketone bodies has been demonstrated to be crucial in tumor progression and metastasis formation, and the high expression of the enzymes required for ketone utilization is predictive of the aggressiveness of tumor cells [10]. 

**Figure 1:**
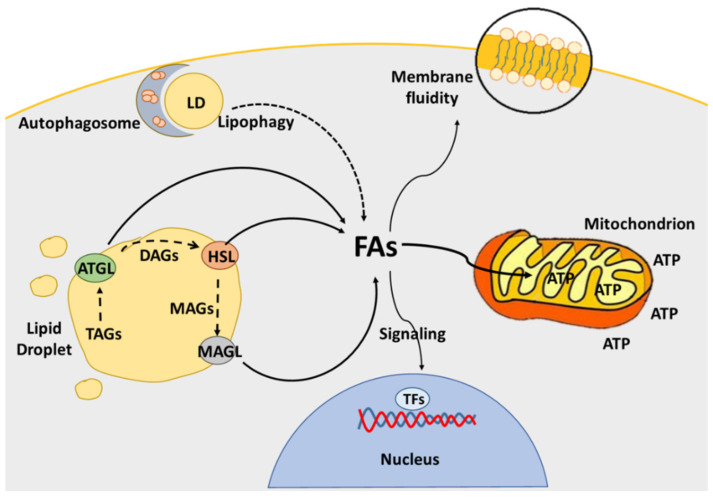
Role of FAs deriving from lipolysis and lipophagy. Besides the energetic role that FAs play when oxidised into mitochondria, they are useful in membrane building block biosynthesis. This function regulates the fluidity of the plasma membrane, which increases during the metastatic process. Moreover, FAs can act as second messengers, activating transcriptional factors and transducing the signal into the nucleus.

Beyond glucose, glutamine and ketone bodies, lipids represent another class of nutrients of which the metabolism is intensely affected in cancer cells [11]. The major source of lipids for normal cells is the exogenous uptake of fatty acids (FAs). In addition to this, cancer cells over-activate the lipogenic pathway to cope with the high demand of FAs necessary in many different contexts [12]. Among their roles, FAs are incorporated into phospholipids acting as building blocks for new membrane formation, which is a needful process, especially for highly replicative cells [13]. As the main constituents of membranes, lipids can also regulate the fluidity of the plasma membrane, contributing to its remodelling, a process exploited by tumor cells during tumorigenesis and metastasis formation [14]. For instance, the content of sphingolipids in membranes is associated with tumor growth and metastasis formation by augmenting the fluidity of lipid bilayers [15,16]. The impact of the enhanced lipid metabolism in cancer is also extended to intracellular signalling. Indeed, lipids can act as second messengers, and can modulate post-translational modifications of proteins [12]. Regarding the canonical role of FAs, namely fueling energy production, it has largely been demonstrated that intracellular lipid droplets provide FAs that are funnelled to mitochondria for β-oxidation and energy purposes [17] (Figure 1). In many types of cancer, this metabolism plays a key role in allowing cancer cells to thrive in a harsh environment, even under the scarce availability of oxygen [18,19]. 

Based on the key role of metabolism in supporting cancer cell proliferation, several efforts have been made to search for strategies to target it, with the aim of improving the current anti-tumor therapies and restraining drug resistance [20]. However, tumor cell metabolism heterogeneity is the main problem to solve in the development of new treatments, and this can be exploited through a deeper understanding of the complex energetic scenario that characterizes the different cancer types, as well as the different cells of the same tumor histotype. 

This review will focus on FA catabolism, a canonical and advantageous process to produce energy through mitochondrial metabolism, which can be central in the heterogeneity of cancer cells’ metabolism due to the concomitant production of reactive oxygen species (ROS). Indeed, given that mitochondria are the main source of intracellular ROS, cancer cells that rely on OXPHOS are exposed to their detrimental effects, and consequently need to be differently equipped in order to cope with their toxicity.

## 2. Lipid Metabolism in Cancer

### 2.1. Exogenous and Endogenous Sources of Lipids

The rewiring of lipid metabolism in cancer is extended to several processes, including FA transport, de novo lipogenesis, β-oxidation and lipid storage alterations [12]. Regarding FA uptake, several FA transporters have been found to be up-regulated in cancer. For instance, the expression of cluster of differentiation 36 (CD36), a transporter of long-chain FAs, is associated with cancer aggressiveness and the poor prognosis of several tumor types, such as breast cancer [21], oesophageal squamous cell carcinoma [22] and glioblastoma [23]. The up-regulation of other FA transporters, including Fatty Acid Binding Proteins 3 (FABP3) and 7 (FABP7), instead contributes to tumor growth and survival in hypoxia-reoxygenation [24]. 

The crucial role of lipids in supporting tumor growth also emerges from the observation that metastatic cells preferentially take root in the anatomical vicinity of adipose tissue [25]. Indeed, adipocytes activate lipolysis in order to produce and secrete free FAs, which are taken up by cancer cells [26,27]. In ovarian cancer, adipocytes-derived FAs activate the AMP-activated protein kinase (AMPK) pathway in cancer cells, increasing β-oxidation. The metabolic switch towards β-oxidation is regulated by the inhibitory phosphorylation of acetyl-CoA carboxylase (ACC) and, consequently, the activation of carnitine palmitoyltransferase 1 (CPT-1) and acyl-CoA dehydrogenase, the rate-limiting enzyme regulating the mitochondrial import of FAs and the first enzyme of the β-oxidation pathway, respectively [26]. Although cancer-associated adipocytes can play a fundamental role in the homing of metastasis, besides FAs, other metabolites can sustain metastatic cells, depending on the contexts. For instance, brain metastases originating from different primary tumours (e.g., breast cancer, non-small-cell lung cancer) show a specific adaption to use acetate as a source of energy. Mashimo and colleagues demonstrated that metastatic cancer cells which have taken root in the brain use acetate to feed the TCA cycle by exploiting their up-regulation of acetyl-CoA synthetase enzyme 2 to convert acetate into acetyl-CoA [28]. Intriguingly, the different tropism has been associated with metabolic similarities between cells of the primary tumor and the host tissue [29].

Besides the uptake of FAs, cancer cells can obtain lipids by lipogenesis, which is normally restricted to hepatocytes and adipocytes [30]. In cancer cells, the de novo synthesis of FAs can be responsible for 93% of triacylglycerols [31]. A great deal of research in the field has shown that lipogenesis can stimulate the cell division of cancer cells, as is also supported by the positive correlation between fatty acid synthase (FAS) and tumor development [32,33]. FAS expression induces the progression of cervical cancer cells into the S phase of the cell cycle, and its inhibition can improve the effects of chemotherapy [34]. In breast cancer, the up-regulation of several enzymes involved in lipogenesis, among which is FAS, is associated with higher cell migration and the repression of apoptosis [31,35,36]. Also in gliomas, the high energy demand is supplied by an increased expression of FAS, which is responsible for the prominent accumulation of lipids in these tumors in comparison to normal brain tissue. In particular, in glioblastoma, the enhanced lipogenesis is a specific feature of cancer stem cells, contributing to maintain the stemness by feeding lipid catabolism. For this reason, despite the increased production of lipids, cancer stem cells have decreased levels of neutral lipids, which allow us to distinguish them from non-cancer stem cells [37]. 

Therefore, the increase of lipogenesis represents a part of the energetic balance which aims to sustain hyper-proliferative cancer cells. Indeed, in the cancer context, the rate of glucose catabolism exceeds the bioenergetic request; thus, the last product of glycolysis, pyruvate, can be redirected toward de novo FA synthesis in order to maintain the constant lipid availability [31]. 

Although in standard growth conditions glucose is the main source of carbons for fatty acid synthesis, under stress, such as hypoxia or mitochondrial impairment, the reductive metabolism of glutamine can be preferred. This metabolism consists of the conversion of glutamine into α-ketoglutarate and, subsequently, it is converted into isocitrate by IDH1, to be funnelled into the fatty acid synthesis pathway [7,38].

### 2.2. The Autophagic Degradation of LDs: Lipophagy

As a mechanism to produce reserves, most lipids are funnelled to the lipid droplets (LDs), cytoplasmic organelles characterized by a phospholipid monolayer surrounding the triglyceride [39]. LDs are lipid reservoirs exploited to store energy and to prevent lipotoxicity [40,41]. Indeed, LDs are a mechanism to buffer FA excess, and are particularly prone to be targeted by ROS and converted into toxic reactive lipids [40]. Thanks to their dynamism, LDs integrate lipid storage and usage, as they can be remodelled by different LD-associated proteins, including lipases, and by lipophagy, the autophagic degradation of LDs [42]. Moreover, LDs represent a crucial step in the uptake and usage of exogenous lipids, which must be stored in LDs before they can be metabolized by the cell. This also occurs for FAs derived from the surrounding adipocytes, in which FAs are stored inside LDs before they feed mitochondrial metabolism to energetically sustain the cells [27]. The mobilization of LD-derived lipids is particularly important in stress conditions, such as nutrient restriction, during which lipid catabolism ensures an efficient and rapid energy supply to cancer cells thanks to the autophagic degradation of LDs [24,43,44]. Lipophagy represents an alternative mechanism to access lipid stores mediated by the interaction between autophagic mediators (e.g., LC3) and proteins located on LDs possessing LC3-interacting (LIR) domains [43]. For this reason, many lipases (e.g., ATGL, PNPLA8) have one or more LIR domains [45,46]. After LD has been trapped into the autophagosome, it is channeled into the macroautophagy pathway, and its degradation takes place in lysosomes by acid lipases [43]. Beyond the rapid lipophagic process, the mobilization of the lipids contained in the LDs occurs through an enzymatic process mediated by lipases, namely lipolysis. This process is responsible for the release of FAs, which can feed mitochondrial metabolism or can be used as signaling molecules that activate transcription factors, such as those belonging to the peroxisome proliferator-activated receptor (PPAR) family [40,42]. Lipolysis is particularly important to maintain intracellular homeostasis, avoiding apoptosis, as the huge accumulation of neutral lipids in non-adipose cells represents a death stimulus [47]. Based on this knowledge, LD dynamism and, in particular, lipolysis have been deeply studied in the cancer context for their multifaceted role in modulating oxidative metabolism, intracellular ROS content, mitochondrial biogenesis and stress-responsiveness [18,40,48,49,50]. 

### 2.3. Lipid Catabolism 

The enzymes that catalyze the FA release from LDs are cytoplasmic lipases: adipose triglyceride lipase (ATGL) is the first enzyme of the lipolytic cascade due to hydrolysing TAGs in diacylglycerols (DAGs) and free FAs. Hormone-sensitive lipase (HSL) catalyzes the hydrolysis of DAGs into monoacylglycerols (MAGs) and free FAs, and, in turn, monoacylglycerol lipase (MAGL) hydrolyses MAGs into free glycerol and FAs [51] (Figure 1). ATGL is the rate-limiting enzyme of the lipolytic cascade, as its expression and functionality regulate the amount of FAs either channelled into mitochondria for β-oxidation or used as second messengers for transcription factor activation [48]. Alterations of ATGL expression have been found in several tumors, in which it can contribute to the satisfaction of the energy request, activating FA-responsive transcription factors and maintaining lipid homeostasis [52,53,54,55]. However, the findings did not evidence an expression pattern in cancer: the overexpression of ATGL was described in high-grade tumors, among which were pancreatic adenocarcinoma [56] and cervical cancer [57], whereas the reduction of ATGL expression was observed in liver and lung cancer [50,58]. In breast cancer, the increase of lipid storage and its oxidation in mitochondria reflects the pro-tumoral support of the microenvironment, as shown by mapping the fate of FAs deriving from the surrounding adipocytes. Coherently, breast cancer cells exposed to adipocyte-conditioned media show enhanced lipolysis and FAs oxidation, which are responsible for the increased proliferation rate. As a consequence, HSL/ATGL knockdown attenuated the cancer cell response to the proliferative stimuli deriving from surrounding adipocytes [59].

However, the breast cancer response to ATGL modulation is quite heterogeneous, because it depends on several factors, among which is the cancer cell density. Indeed, a low cell density promotes lipid catabolism via ATGL, fuelling mitochondrial β-oxidation and producing ROS, which sensitizes cells to ferroptosis triggered by lipid peroxidation. Instead, in high-density conditions, the down-regulation of ATGL and, consequently, FA β-oxidation confer cancer cells with resistance to ferroptosis [60]. 

In hepatocellular carcinoma, ATGL is found to be down-regulated, and its forced expression reduces cancer cells’ proliferation by activating PPARα through FAs generated by lipolysis [50]. In this context, FAs deriving from ATGL activity act as signaling molecules that promote the switch from glycolysis to OXPHOS. Xie et al. demonstrated that ATGL-mediated lipolysis decreases the proliferation of five different cancer cell lines, suggesting low ATGL activity as a marker of tumor aggressiveness [61]. Overall, these opposite roles of ATGL in the different tumor types could suggest a tissue-dependent effect, consequently, on the different pathways in which ATGL-derived FAs could be funnelled. 

Despite most papers regarding the rate-limiting enzyme of lipolysis to be ATGL, MAGL and HSL have also been associated with tumorigenesis. MAGL has been unveiled as a tumor promoter, being highly expressed in aggressive human cancer cells. This effect is recapitulated after MAGL over-expression in non-aggressive cancer cells, and it is reversed by inhibiting MAGL in aggressive ones [62]. Regarding HSL, it is down-regulated in pancreatic cancer by the oncogene KRAS, which is responsible, consequently, for the increase of lipid storage. The disruption of the KRAS/HSL axis leads to an inhibition of the invasive migration and the metastatic phenotype [63]. 

Lipid catabolism can also sustain tumors through lipophagy activation, which allows cancer cells to rapidly access the lipid reserves. This phenomenon has been observed in hepatocellular carcinoma, in which lipophagy promoted the resistance to starvation and carcinogenesis, predicting a poor patient prognosis [64]. In lung cancer, the key role of lipophagy in cell survival during starvation has been demonstrated by the impairment of fatty acid oxidation (FAO) in Autophagy-Related 7 (ATG7)-deficient lung tumors, which also show increased sensitivity to FAO inhibitors and a defective capacity to use lipid stores [65]. It has been demonstrated that glioblastoma cells also cope with glucose starvation by augmenting the TAG utilization by lipophagy. This high plasticity of tumor cells allows them to quickly hydrolyse LD storage, realising a huge amount of energy, which can be used to repair drug damages in the case of tumor resistance [66]. Similarly to lipolysis, lipophagy can also play opposite roles depending on the tumor context. For instance, in HeLa cells, increasing lipophagy by the over-expression of ATG14 leads to the accumulation of FAs resulting from the accelerated degradation of LDs that boost mitochondrial metabolism and ROS-dependent apoptosis [67]. 

In those tumor types in which lipid catabolism showed pro-tumoral functions, this pathway has been evaluated as a potential therapeutic target. The pharmacological inhibition of the CPT-1 enzyme, which allows the entry of long-chain fatty acids into mitochondria for oxidation, has been identified as an approach for decreasing the cancer cell proliferation in prostate cancer [68], human leukaemia cells [69], triple-negative breast cancer [70] and glioma [71]. Intriguingly, Singh and colleagues demonstrated that the CPT-1 inhibitor etomoxir kills cancer stem cells without side effects on normal cells, providing clear-cut evidence of cancer’s addiction to the lipid catabolism pathway [72]. This approach could have a double positive effect, considering that FAO is the energetic pathway preferentially used by tumor-associated macrophages (TAMs) for energy supply [73]. β-oxidation has been also indicated as a contributing factor in the differentiation process of macrophages into the M2 phenotype, which is similar to TAMs. The mechanism underlying the M2 phenotype seems to rely on the mitochondrial oxidation of FAs and ROS production, which contribute to the reprogramming toward the TAM phenotype by activating STAT6 [74].

Moreover, the alteration of lipid catabolism is related to the possibility to exploit it as a predictive or diagnostic marker. Regarding this, in many cancers, including bladder cancer, several metabolites involved in β-oxidation have been differentially detected in the metabolomic profile of the biological fluids of cancer patients with respect to healthy controls and, for this reason, they have been suggested as diagnostic markers of the tumor [75]. 

## 3. Lipid Catabolism Regulates Intracellular ROS and Vice Versa

### 3.1. Lipid Catabolism as a Source of ROS in Cancer Cells 

Many effects of lipid catabolism are the consequences of ROS production, because the mitochondrial electron transport chain is the main place where they are produced. In vitro experiments show that the main complexes responsible for ROS production are I, II and III, although the measurement of the ROS amount produced by each respiratory complex has a low resolution in vivo. Complex I and II produce ROS in the mitochondrial matrix, whereas complex III releases ROS in both the matrix and intermembrane space [76]. ROS production is due to the premature leak of electrons from the abovementioned complexes, which causes the reduction of oxygen to superoxide [77]. Increased FA availability, upon lipase activation, exacerbates the rate of electron flux with a concomitant increase in superoxide anions and hydrogen peroxide formation [78,79]. Therefore, the use of FAs and the increased ROS flux are interrelated, and add new players in the tumor growth regulatory process [78]. 

**Figure 2:**
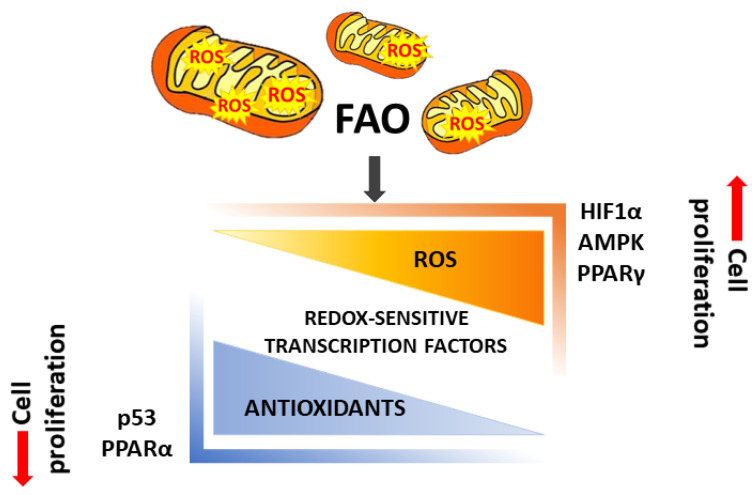
The balance between ROS production and elimination contributes to the regulation of tumor cell proliferation. FAO is responsible for mitochondrial ROS production, which in turn is buffered by intracellular antioxidants. Downstream of FAO, the ROS are low in tumor cells equipped with prominent antioxidant systems, whereas the ROS are high if not properly buffered. In this case, ROS are able to activate redox-sensitive transcription factors (e.g., HIF1α, AMPK, PPARγ), inducing cell proliferation. Instead, more efficient antioxidant systems allow FAs to play a signaling role, activating selective transcription factors (e.g., PPARα, p53), reducing cell proliferation.

ROS can participate in several cancer pathways implicated in tumor growth, metabolism, differentiation, inflammation and metastasis [80]. They can act as second messengers, regulating the targets by reversible oxidations. These highly active radicals, which can derive from intrinsic or extrinsic sources, are able to trigger signaling cascades led by protein kinases, phosphatases and transcription factors, including the mitogen-activated protein kinase (MAPK) cascades, phosphoinositide-3-kinase (PI3K)/Akt and nuclear factor kappa-light-chain-enhancer of activated B cell (NF-kB) pathways [80,81,82,83,84]. Given that cancer cells have higher levels of intracellular ROS than normal cells [85], the balance between ROS production and elimination is particularly crucial in regulating their survival [86] (Figure 2). Indeed, even if low levels of ROS can promote tumor proliferation, intracellular ROS accumulation can cause oxidative damage to macromolecules, such as DNA damage, lipid peroxidation and protein oxidation [87,88]. In light of this, the accumulation of lipids in LDs plays also a role in maintaining intracellular redox homeostasis, avoiding the oxidative stress that would derive from lipid catabolism [89]. For instance, in glioblastoma, a tumor which ensures its growth through the acquisition of a large amount of FFAs, the enzyme diacylglycerol-acyltransferase 1 (DGAT1) is responsible for the storage of excess FAs in LDs. The inhibition of this enzyme leads to an excessive β-oxidation, which results in a detrimental production of ROS and apoptosis [90]. On the other hand, disrupting the redox balance by increasing β-oxidation represents a strategy exploited by several tumor suppressors, including PPARγ, to induce apoptosis [91,92]. Besides mechanisms aiming to avoid ROS production, cancer cells cope with the threat induced by ROS through antioxidant systems, which participate in cancer progression and therapy resistance by buffering ROS [93,94,95,96]. In line with this, the antioxidant capacity contributes to the cancer-specificity effect of the lipolytic pathway, regulating the response of cancer cells to modulations of lipid catabolism [57,97,98,99]. In cervical cancer cells, we demonstrated that boosting lipid catabolism by ATGL over-expression enhances the proliferation and the “Warburg effect”. The increased intracellular ROS content was directly related to mitochondrial FA oxidation downstream of the higher rate of lipolysis. In turn, ROS mediate the metabolic switch by up-regulating hypoxia-inducible factor-1α (HIF-1α), a well-known promoter of glycolysis [57]. Despite the fact that the increase of glycolysis appears as an inconsistent adaptation to the boosted mitochondrial metabolism, the involvement of mitophagy deciphered the mechanism through which cancer cells manage intracellular ROS content. Mitophagy represents a key process for maintaining redox homeostasis, on which depends the cancer cell fate in ROS-inducing conditions, such as a rise of lipid catabolism [100]. This notion correlates with what has been demonstrated in cervical cancer, in which the pro-tumoral effect of the lipolytic rise turns anti-tumoral when mitophagy is blocked. Indeed, cancer cells exploit mitophagy as a survival mechanism to remove ROS-damaged mitochondria, whereas, when the mitophagy is inactivated, ROS accumulation causes cancer cell death [57]. The ROS tolerance threshold, beyond which cells die and below which signals promoting the tumor are triggered, is largely dependent on the cancer cell type and tumor microenvironment conditions (e.g., oxygen and nutrient availability, inflammation, and vascularization) [101]. For instance, in the mouse model of mammary tumorigenesis, the blockage of BNIP3-mediated mitophagy has an opposite effect with respect to that described in cervical cancer [102]. The higher ROS content in tumor cells became an interesting intervention point of many therapeutic approaches, which exploit this feature specifically to kill cancer cells by generating a redox imbalance. Indeed, several anticancer approaches employ pharmacological ROS-inducing agents or metabolic alterations, aiming to turn this cancer hallmark into a vulnerability by inducing fatal oxidative damage [103]. 

However, the consequent adaptation of cancer cells to high intracellular ROS levels equips them with a higher antioxidant capacity than normal cells. The antioxidant capacity of the different tumor types is hugely heterogeneous. Even different subtypes of the same tumor present a different capacity to maintain the redox balance, as demonstrated in breast cancer [104] and in ovarian cancer, in which the measurement of antioxidants predicts the chemotherapy resistance capacity of the different clones [94]. Therefore, understanding the redox response of cancer cells has crucial therapeutic implications, because the same intervention could have different effects depending on the cellular capacity to restore the redox balance [105]. For instance, boosting lipid catabolism in cervical cancer [57], in highly aggressive breast cancer cells [106] and in pancreatic cancer [107] is responsible for a more aggressive phenotype due to increasing intracellular ROS. On the contrary, in hepatocellular carcinoma has anti-tumoral consequences, without effects on the intracellular ROS content [50].

### 3.2. ROS Can Modulate the Lipid Availability in Cancer Cells 

The intricate relationship between lipid catabolism and ROS in cancer cells is further widened by the capacity of radicals to regulate lipid metabolism. Many lines of evidence have demonstrated that different prooxidant conditions result in lipid accumulation in cancer cells as a defense mechanism to avoid the exacerbation of oxidative stress [108]. In this context, ROS act as second messengers to block lipolysis, inducing negative feedback in which the ROS play a self-limiting role [109]. They transduce the signal into the nucleus by the activation of several transcription factors, including the abovementioned HIF-1α, which contributes to the reduction of intracellular ROS by suppressing lipolysis. Regarding this, Zhang et al. demonstrated that tumor cells adapt to hypoxia and reduce ROS deriving from FAO blocking ATGL activity via hypoxia-inducible gene 2 (HIG2), a HIF-1 target, funnelling TAGs into LDs [48,110]. In liver cancer cell lines, ROS mediate the activation of HIF-1α, which is responsible, in turn, for suppressing long-chain acyl-CoA (LCAD) and medium-chain acyl-CoA dehydrogenase (MCAD) expression as a mechanism decreasing FAO [78]. In vitro experiments in the HepG2 hepatoma cells highlighted another mechanism through which ROS induce lipid accumulation. Indeed, cells treated with H_2_O_2_ over-express perilipin-2 (PLIN-2), showing as a consequence an increased content of LDs. The results of the in vitro experiments were also recapitulated in vivo, in which H_2_O_2_ injection in mouse livers increases the lipid content [111]. Therefore, ROS, and particularly mitochondrial ROS, are also exploited by cancer cells to reprogram lipid metabolism, with the aim of driving tumor progression. Recently, it has been demonstrated that mitochondrial elongation factor 2 (MIEF2) over-expression drives the progression of ovarian cancer by enhancing lipid accumulation. In this system, MIEF2 induces an ROS-mediated activation of the AKT/mTOR signaling pathway, resulting in an increase of LDs, which are instead reduced by MIEF2 silencing or treatment with the antioxidant N-acetylcysteine (NAC) [112]. Antioxidant treatments have shown the capacity to reactivate FAO, demonstrating the bidirectional relation between lipid catabolism and intracellular ROS content [113]. 

Another factor linking ROS to lipid catabolism is the AMP-activated protein kinase (AMPK). This energy sensor of cells is also activated in stress conditions, such as ROS accumulation, and promotes metabolic reprogramming by inducing lipolysis and FAO [114,115]. Indeed, among the AMPK downstream factors there are ATGL and HSL, which are activated by the phosphorylation of Ser406 and Ser563, respectively [115]. The activation of AMPK also occurs in hepatoma cells upon hepatitis C virus infection, during which it is responsible for an enhanced β-oxidation. In this condition, the ROS-mediated phosphorylation of Thr172 activates AMPK and, in turn, induces several genes involved in the antioxidant response to balance the redox homeostasis and promote survival. The key role of ROS is shown by the evidence that NAC treatment reversed the phenotype [116].

AMPK regulates fatty acid metabolism not only by activating FAO but also by inhibiting FAS through the phosphorylation of acetyl-CoA carboxylase 1 (ACC1) and acetyl-CoA carboxylase 2 (ACC2), which would otherwise block CPT-1 by producing malonyl-CoA. This regulation promotes tumor cell survival during stress conditions by maintaining ATP homeostasis, and by providing NADPH [117]. NADPH is mainly produced by glucose 6-phosphate dehydrogenase (G6PD) and 6-phosphogluconate dehydrogenase (6PGD) in the pentose–phosphate pathway. The cytosolic pool of NADPH is also maintained by three other enzymes, isocitrate dehydrogenase 1 (IDH1), malic enzyme 1 (ME1) and 10-formyltetrahydrofolate dehydrogenase (ALDH1L1). In the mitochondrion, NADPH synthesis is mainly dependent on nicotinamide nucleotide transhydrogenase (NNT), isocitrate dehydrogenase 2 (IDH2), malic enzyme 3 (ME3), mitochondrial homolog of 10-formyltetrahydrofolate dehydrogenase (ALDH1L2) and methylenetetrahydrofolate dehydrogenase 1-like (MTHFD1L) [118]. Although these pathways are the main ones, FAO can contribute to NADPH production by the reaction of acetyl-CoA with oxaloacetate that generates citrate. Citrate exported into the cytoplasm could be used by ME1 and IDH1 to produce NADPH [119]. NADPH is a fundamental coenzyme that is particularly required during oxidative stress to regenerate reduced glutathione (GSH), the most important non-enzymatic antioxidant. GSH plays a fundamental role in ROS detoxification as both a direct ROS scavenger and cofactor of the enzyme glutathione peroxidase (GPx), which is the first-line defence against peroxides. GPx utilizes the reducing power of GSH, which is converted to the di-sulphide-oxidized form (GSSG). GSSG is reduced back to GSH by glutathione reductase, a flavoenzyme that uses NADPH as a cofactor [14]. 

Thus, a controversial relation exists between lipid catabolism and ROS production, in which, on the one hand, the β-oxidation of FAs is responsible for intracellular ROS increase, and on the other hand, FAO is linked to NAPDH and GSH synthesis, contributing to redox homeostasis [109]. This paradoxical effect reflects the complexity of the intricate intracellular balance, which requires a deep knowledge of the cellular heterogeneity in order to be therapeutically exploited. 

For instance, the augmented FAO sustains the progression of RAS-mutated lung and pancreas carcinomas especially through NADPH production, allowing glutathione reductase to restore GSH. The evidence of the dependence on GSH-mediated ROS detoxification is further strengthened by the arrest of its proliferation upon the blocking of glutathione synthesis with buthionine sulfoximine (BSO) treatment [120]. During glucose deprivation and pentose–phosphate pathway impairment, the NADPH pool is preserved by AMPK, and activated by energy stress. Indeed, AMPK phosphorylates ACC1 and ACC2, limiting lipogenesis, which is the main metabolic pathway responsible for the consumption of NADPH. The consequent reduction of malonyl-CoA activates CPT-1, promoting FAO. Regarding this, the authors speculate that the continuous replenishment of TCA cycle metabolites provided by β-oxidation could contribute to the restoration of NADPH [117,121]. For this reason, the failure to activate AMPK upon glucose starvation results in the death of tumor cells, whereas treating AMPK-deficient cells with antioxidant compounds, such as NAC, inhibits death [117]. Although AMPK is the direct sensor of the stimulus, and triggers the metabolic switch, other downstream factors can contribute to maintaining the active FAO previously induced by AMPK, including p53 [122]. 

Among the numerous roles of p53 in cancer, this factor is also able to regulate lipid metabolism upon ROS increase, promoting FAs catabolism and simultaneously preventing lipid synthesis [122,123]. Indeed, p53 facilitates the entry of lipids into the mitochondria by increasing CPT-1 gene expression, and promotes the expression of the Lipin1 (Lpin1) gene, a regulator of lipid metabolism [122,124]. Lpin1, together with peroxisome proliferator-activated receptor-gamma coactivator 1a (PGC-1a) and peroxisome proliferator-activated receptor alpha (PPARα), induces genes promoting FAO and inhibiting FA synthesis.

## 4. Targeting Lipid Catabolism and ROS: New Insights in Cancer Therapy 

### 4.1. Exploting FAO as an Anti-Tumor Strategy 

**Figure 3:**
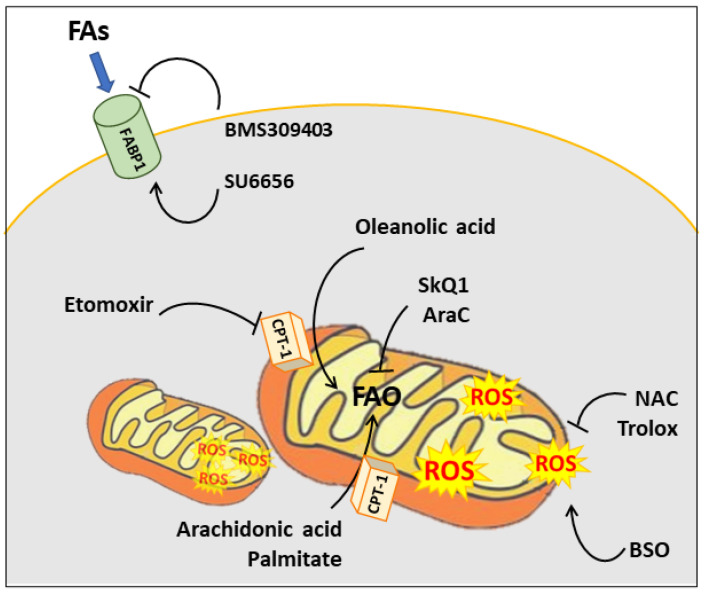
A schematic representation of the mechanism of action exploited by the different compounds mentioned.

The findings related to cancer metabolism have been translated into clinical practice, and many research papers have provided proof that targeting lipid catabolism represents a useful therapeutic approach. Nevertheless, the interconnection between lipid catabolism and intracellular ROS plays a role in the efficacy of treatments. Indeed, modulating β-oxidation, drugs have an indirect effect on ROS, that, if understood, could be fruitfully exploited in different cancer types. Based on cancer cells’ capacity to cope with ROS, radical species can have an adjuvant or limiting effect on drugs targeting lipid metabolism. Numerous approaches take advantage of the prompting of lipid catabolism to increase the ROS levels above the death threshold (Figure 3). 

For example, oleanolic acid is a pentacyclic triterpenoid compound used in cancer therapy to inhibit cancer initiation and progression, and to reduce metastasis formation [125]. It has been demonstrated that this treatment has an inhibitory effect on hepatocellular carcinoma by mediating mitochondrial-dependent apoptosis. In particular, oleanolic acid enhances lipolysis, feeding mitochondrial metabolism, as shown by CPT-1a up-regulation. As a result, it causes an increase in mitochondrial ROS production, which is responsible for mitochondrial apoptosis. The involvement of ROS in the apoptotic cell death mediated by oleanolic acid is proved by treating cells with the mitochondrial-targeted antioxidant MitoQ, which blocks the apoptotic response [126]. Similarly, in rat hepatoma cells, arachidonic acid—a long-chain polyunsaturated fatty acid—induces apoptotic cell death at micromolar concentrations via ROS production. In parallel, the combination of arachidonic acid treatment with Trolox, a tocopherol derivative with potent antioxidant properties, abolishes the pro-apoptotic effect [127]. In another work on rat hepatoma cells, palmitate recapitulates the effect obtained with arachidonic acid, stimulating ROS-dependent apoptotic cell death, which is indeed partially blocked by NAC supplementation [128]. The same evidence has been highlighted on human hepatoblastoma cell lines treated with palmitate, which showed a time-dependent ROS production and a release of hydrogen peroxide that led to the loss of mitochondrial potential, and subsequently to cell death. Consistently, the cytotoxicity of palmitate is counteracted by hydrogen peroxide scavengers [129]. A similar effect is obtained by an Src inhibitor, SU6656, that suppresses lung cancer growth via ROS generated by β-oxidation. Treating cells with SU6656 induces FABP4, facilitating lipolysis and accumulating ROS, which, in turn, lead to apoptosis [92]. 

The relationship between lipid metabolism and oxidative stress is also exploited to produce new advantageous combinations with several canonical cancer treatments to increase their efficacy. Given that cancer cells have elevated ROS levels, many chemotherapeutic drugs induce cancer cell apoptosis by inducing oxidative damage. However, cancer cells evolved antioxidant mechanisms to circumvent apoptosis induction. Based on this, the modulation of intracellular ROS has been considered as an approach to overcome drug resistance, and boosting FAO has shown advantages in this context. For instance, several studies have highlighted that n-3 docosahexaenoic acid (DHA) not only stimulates apoptosis per se [130] but also increases the responsiveness of cancer cells to doxorubicin by generating ROS [131]. It has also been proved that treatment with n-3 fatty acids sensitizes leukemic cell lines to doxorubicin, vincristine and fludarabine, owing to an increase of ROS [132]. 

### 4.2. The Other Side of the Coin: The Pro-Tumor Effects of ROS Produced by Boosted FAO

This tricky scenario is further complicated by the cell-specific role of ROS. Recent works have highlighted that β-oxidation is an up-regulated pathway in cancer stem cells, in which it contributes to sustaining cancer metastasis and the epithelial-to-mesenchymal transition by producing ROS [133]. This feature represents a cell-intrinsic drug resistance mechanism, and, for this reason, strategies aiming to target mitochondrial ROS homeostasis have been considered [134]. This heterogeneity mirrors the cell-dependent role of radical species, which can act as signaling molecules linking FAO to the metastatic phenotype. Therefore, the pharmacological inhibition of FAO in combination with mitochondrial-specific agents, such as SkQ1, has been proposed as a promising therapeutic approach [133,135]. In line with this, the augmented FAO is disclosed in acute myeloid leukaemia cells as a mechanism to develop cytarabine (AraC) and mitoxantrone resistance. Intriguingly, the resistant cells showed a higher OXPHOS and ROS content than the sensitive ones. Thus, treatment with several pharmacological agents inhibiting OXPHOS (tigecycline, ethidium bromide, phenformin, metformin, rotenone, or antimycin A) in association with AraC and mitoxantrone significantly decreases their EC_50_. More specifically, the metabolic plasticity of AraC and mitoxantrone residual cells was assessed, highlighting that mitochondrial metabolism was fed by FAs. In light of this, these drugs were tested in combination with etomoxir, disclosing a useful mechanism of chemosensitization [136,137]. A similar mechanism has been identified in residual breast cancer cells responsible for tumor recurrence, because they show a metabolic shift, with increased FA transport into mitochondria and high ROS levels. It has been demonstrated that antioxidants, such as NAC, induce a significant reduction of tumor recurrence in mice. In this study, antioxidants are proposed as an adjuvant therapy bestowing a survival advantage; the concept is also supported by the fruitful use of the dietary supplementation of vitamins C and E during the period following a breast cancer diagnosis [138]. 

Similarly, a pro-survival effect of β-oxidation and ROS production has been identified after the transfer of lipids from adipocytes surrounding a tumor mass to cancer cells, which can store or use lipids to produce energy. In several types of tumors, the increased mitochondrial metabolism deriving from the relationship with adipocytes and the augmented ROS production make cancer cells more tumorigenic [139,140,141]. Consistently, the inhibitors of transporters mediating lipid uptake have been employed as anti-cancer compounds. Among them, BMS309403—an inhibitor of FABP4—has shown promising effects on ovarian cancer [139].

Another mechanism favouring tumorigenesis via β-oxidation is NADPH production, through which cancer cells replenish the GSH pool, controlling their redox state and avoiding oxidative stress. Indeed, an elevated GSH level is a marker of tumor aggressiveness and drug resistance [142]. As is consistent with this knowledge, several works show that etomoxir is also useful in cancer treatment to trigger an unbalance of the redox state, which leads tumor cells to death [119,143,144]. As a consequence of a prominent FAO, this type of tumor is more sensitive to BSO, which leads to an overproduction of ROS by removing the GSH-based redox buffering capacity. For instance, many papers have demonstrated that GSH depletion sensitizes cisplatin- and temozolomide-resistant cells [145,146]. Moreover, *RAS*/*REDD1* mutant tumors, mainly supplied by the augmented FAs uptake, have shown a critical sensitiveness to BSO treatment, which induces growth arrest in vivo [120]. A representative list of the ROS-mediated effects of compounds is reported in Table 1. 

## 5. Conclusions

Despite the fact that it is now widely known that cancer cells display a reprogrammed lipid metabolism, the different consequences observed upon lipid catabolism modulation are still unclear. Beyond the numerous roles that lipids can play in cancer cells, they are also responsible for mitochondrial ROS production, which, in turn, can act as signaling molecules implicated in several pathways. Cancer cells need the fine-tuned regulation of the respiratory chain complex, antioxidant response and detoxification capacity. Therefore, cancer cells that display a dual capacity for glycolytic metabolism and OXPHOS easily direct metabolic flows by becoming more aggressive. On the other hand, the close interaction between FAO and ROS provides useful intervention points to ameliorate the effectiveness of therapy and restrain drug resistance. Lipid catabolism can be exploited as a metabolic trick to augment ROS, increasing the sensitivity of cancer cells to anticancer compounds, as has been demonstrated for other metabolic rearrangements promoting mitochondrial metabolism, including ketone bodies [148,149]. On the other hand, based on the evidence that several tumors take advantage of ROS deriving from β-oxidation to become more resistant to drugs, interventions aiming to reduce mitochondrial metabolism and ROS generation are considered to improve the success of therapy in those cases. In the end, the antioxidant capacity of cancer cells is still crucial to determine whether ROS will act as signaling molecules or represent cancer cells’ Achille’s heels. In light of this mutual relationship, the founding mechanisms and features that can be exploited in the prediction of the cancer cell response to lipid metabolism alterations are still an open question, and are promising for research in metabolic cancer cell adaptation. 

## Figures and Tables

**Table 1:** List of studies evaluating the effects of compounds inducing FAO and the standard therapy combination in tumor treatment.

Drug	Tumor	Effect	References
Oleanolic acid	Hepatocellular carcinoma	Oleanolic acid enhances lipolysis stimulating ROS production, which is responsible for mitochondrial apoptosis.	[125]
Arachidonic acid	Rat hepatoma cells	Arachidonic acid-induced ROS leading cells to apoptosis.	[127]
Trolox	Rat hepatoma cells	Trolox prevents arachidonic acid-induced apoptosis buffering ROS	[127]
Palmitate	Rat hepatoma cells Human hepatoblastoma	Palmitate stimulates mitochondrial metabolism and ROS production, leading cells to apoptotic death	[128,129]
N-acetyl cysteine (NAC)	Rat hepatoma cells Breast cancer	NAC limits ROS accumulation in palmitate treatment reducing apoptotic and enhancing cell viability.	[128,138]
SU6656	Lung cancer	SU6656, an Src inhibitor, results in FABP4 induction. The consequent activation of β-oxidation generates ROS that induces apoptosis	[92]
Docosahexaenoic acid (DHA)	Prostate cancer Breast cancer Leukaemia	DHA generates ROS, consequently activating apoptosis. The co-treatment of DHA with other drugs increases the responsiveness to these.	[130]
SkQ1	Cancer stem cell	SkQ1 is a mitochondrial-specific antioxidant in combination with the pharmacological inhibition of FAO has been proposed as a promising therapeutic approach	[133,135]
Cytarabine (AraC)	Leukaemia	AraC treatment causes an increase of the OXPHOS with higher ROS content. This aspect is useful to increase the sensitivity to other drugs, including etomoxir.	[136,137]
Vitamin C/E	Breast cancer	The introduction of vitamins C and E in the diet confers a protective effect from tumor recurrence and survival of patients.	[138]
BMS309403	Ovarian cancer	BMS309403 is an inhibitor of FABP4 that impacts cell proliferation affecting β-oxidation and ROS production.	[139]
Etomoxir	Glioblastoma Leukaemia	Etomoxir is a CPT1a inhibitor that affects the redox homeostasis causing the decrease of NADPH level and thus the GSH content leading cells to death. An off-taget effect of Etomoxir is due to the oxidative stress caused by inhibiting the mitochondrial adenine nucleotide transporter and complex Ι of the electron transport chain.	[143,144,147]
Buthionine sulfoxamine (BSO)	Lung cancer Pancreatic cancer	BSO is an inhibitor of the GSH synthesis that leads to an overproduction of ROS by removing glutathione-based redox buffering capacity.	[120]
